# Review on Genomic Regions and Candidate Genes Associated with Economically Important Production and Reproduction Traits in Sheep (*Ovies aries*)

**DOI:** 10.3390/ani10010033

**Published:** 2019-12-23

**Authors:** Gebremedhin Gebreselassie, Haile Berihulay, Lin Jiang, Yuehui Ma

**Affiliations:** 1 Key Laboratory of Animal (Poultry) Genetics Breeding and Reproduction, Ministry of Agriculture, Institute of Animal Science, Chinese Academy of Agricultural Sciences (CAAS), Beijing 100193, China; gerageruggg@gmail.com (G.G.); haile.berihulay@yahoo.com (H.B.); jianglin@caas.cn (L.J.); 2 National Germplasm Center of Domestic Animal Resources, Institute of Animal Science, Chinese Academy of Agricultural Sciences (CAAS), Beijing 100193, China; 3 Department of Agricultural Biotechnology, Biotechnology Center, Ethiopian Biotechnology Institute, Ministry of Innovation and Technology, Addis Ababa 1000, Ethiopia

**Keywords:** sheep, candidate gene, genomic region, phenotype trait, *Ovis aries*

## Abstract

**Simple Summary:**

Sheep is one of the most economically important animals used as a source of meat, milk, wool, and fur for human society. These commodities are essential for human being. Body growth, body weight, carcass quality, fat percent, fertility, milk yield, wool, horn type and coat color are essential and useful sheep traits. Understanding the genetic background of these traits is paramount to increase the production and productivity of domestic animals. The availability of genomic data, development of molecular breeding techniques, and genome technologies have come to play a vital role in understanding the genetic background of different animal traits. This is directly or indirectly helpful for the practice of genetic improvement of economically important traits in sheep. The identification of genomic regions, genes associated with phenotypic traits, and description of gene function are some of the applied research activities to understand the genetics of livestock species. The aim of this review is to discuss and summarize different reported research findings on identified genomic regions and candidate genes related with economically important traits as well as gene annotation in sheep.

**Abstract:**

Sheep (*Ovis aries*) is one of the most economically, culturally, and socially important domestic animals. They are reared primarily for meat, milk, wool, and fur production. Sheep were reared using natural selection for a long period of time to offer these traits. In fact, this production system has been slowing the productivity and production potential of the sheep. To improve production efficiency and productivity of this animal through genetic improvement technologies, understanding the genetic background of traits such as body growth, weight, carcass quality, fat percent, fertility, milk yield, wool quality, horn type, and coat color is essential. With the development and utilization of animal genotyping technologies and gene identification methods, many functional genes and genetic variants associated with economically important phenotypic traits have been identified and annotated. This is useful and presented an opportunity to increase the pace of animal genetic gain. Quantitative trait loci and genome wide association study have been playing an important role in identifying candidate genes and animal characterization. This review provides comprehensive information on the identified genomic regions and candidate genes associated with production and reproduction traits, and gene function in sheep.

## 1. Introduction

Domestic sheep (*Ovis aries*) is an economically and culturally important farm animal. Sheep are selected mainly for meat, milk, wool and fur production at commercial farm and small holder farmer level. These commodities are economically important for human being [1]. Sheep researchers and scientists have been conducting different problem-solving researches to increase the pace of genetic gain and to prioritize genetically superior animals using artificial insemination and quantitative trait loci (QTL). A QTL is a locus that is associated with phenotypic variation in an organism [2]. In the 1990s, identification of genes associated with different complex traits has been started in domestic animals. In connection with this, approach like the use of QTL have been preferred tool for years to identify economically important phenotypic traits [3]. Although a lot of QTLs for phenotypic traits have been detected using microsatellite markers, the resolution of the map and confidence interval is very low. The reason behind the low resolution power is that QTL usually covers large genomic region (several mega-bases or whole chromosome) which is directly affecting the gene detection potential [3]. Genome-wide association study (GWAS) was developed to fill this gap for phenotypic traits in livestock. GWAS is hypothesis-free method to identify associations between genetic regions and phenotypic traits. It is preferred due to the power it has to detect causal variants and the ability in defining narrower genomic regions [4]. These approaches have been working on the basis of single-nucleotide polymorphisms in different domestic animals [5]. GWAS is an important approach to identify candidate genes and molecular variants associated with different phenotypic traits. This will be quite useful for genetic improvement program of sheep and other animals as well. The 1st successful result of GWAS was a study on myocardial infarction [6], and next was reported on patients with age-related macular degeneration [7]. In sheep, GWAS was performed for the first time in order to understand the molecular background of horn types [8]. Sequencing technologies and bioinformatics are also among the tools used to understand the genetic background, and to elucidate the associations between genes and phenotypic traits in mammals [9].

Up to date, several genes associated with different economically important phenotypic traits and their functions have been reported in domestic animals using different molecular approaches. The aim of this review is to summarize the identified genomic regions and candidate genes associated with different production and reproduction traits (Body growth, body weight, carcass quality, fertility, milk yield, wool, horn type and coat color) in different sheep breeds.

## 2. Genetics and Genomic Studies on Phenotypic Traits in Sheep

### 2.1. Genes Associated with Growth and Body Weight

Growth traits are important economic traits in sheep. The development of molecular genetics and genomic tools opened an opportunity to identify and annotate functional genes associated with economic traits. Although growth and reproduction are regulated centrally by pituitary gland [10], there are also transcription factors and inducing signals that affect the development of pituitary gland. For instance, LIM Homeobox 3 (*LHX3*) and LIM Homeobox 4 (*LHX4*) serve as a transcription factors for sheep body growth and reproduction process [11,12]. A PCR study has identified genetic variants in ovine Calpain (*CAPN*) gene which are related with meat tenderness and growth performance traits in sheep [13,14,15]. Calpains (CAPN) encodes intracellular calcium-activated cysteine proteases that work in physiological and pathological processes. It is responsible for remodeling proteins that maintain the structure of skeletal muscle [16,17]. Another PCR study has reported significant genetic variants in Myocyte enhancer binding factor 2B (*MEF2B*) and Thyrotropin releasing hormone degrading enzyme (*TRHDE*) genes affecting body weight and growth in sheep [18,19,20]. For instance, SNPs have been detected in *MEF2B* and *TRHDE* genes associated with sheep body weight and chest girth [20]. *MEF2B* is a gene that regulates muscular growth and development of sheep. It is part of the myocyte enhancer factor-2 (*MEF2*) gene family (*MEF2A*, *MEF2C*, and *MEF2D*) in vertebrate animals. *TRHDE* encodes a member of peptidase M1 family, and it inactivates the neuropeptide thyrotropin-releasing hormone [20].

In addition to growth and body weight traits, body mass index and body size traits are also an important sheep production traits. A genome wide specific selection study has identified Fat mass and obesity-associated protein (*FTO*) and Apolipoprotein B Receptor (*APOBR*) genes affecting body mass index in German mutton merino, CMF, and African white dorper sheep breeds [18]. *FTO* is part of the alpha-ketoglutarate-dependent hydroxylase gene family, which is a non-heme iron-containing protein. Another gene, Tumor protein p53 (*TP53*) has been identified related with body size in Frizarta sheep. This gene plays a significant role in DNA-binding transcription factor activity and apoptotic process [19]. Haplotype (*DRB1* *2001) is also found related with increase weaning weight, mature weights and average daily gain as well [21]. A PCR-RFLP study has reported genetic variants in myostatin (*MSTN*) gene associated with muscling and growth in Madras Red, Mecheri, and Texel sheep breeds. *MSTN* gene serves as a negative regulator for muscle growth. Thus, muscle growth increases by double when the function of *MSTN* deteriorated in sheep [22,23]. The gene, growth hormone (*GH*) has been identified affecting post-weaning gain. It induces body growth and also reduces fat stores in sheep [24,25].

Furthermore, a genome-wide association study has identified candidate genes associated with post-weaning gain at chromosome-wise level in different sheep breeds. These genes include glutamate metabotropic receptor 1 (*GRM1*), methyl-CpG-binding domain protein 5 (*MBD5*), ubiquitin protein ligase E3 component n-recognin 2 (*UBR2*), ribosomal protein L7 (*RPL7*) and structural maintenance of chromosomes 2 (*SMC2*) [26]. *SMC2* is part of the SMC family genes, and is critical for DNA repair in mammals [27], whereas *GRM1* is one of the metabotropic class of glutamate receptors gene members. The function of *GRM1* is activating phospholipase C (PLC) and releasing glutamate from the presynaptic cell. *UBR2* is a gene encodes an E3 ubiquitin ligase of the N-end rule proteolytic pathway. It targets proteins which destabilize N terminal residues for polyubiquitylation and proteasome mediated degradation. *MBD5* is a gene consists of approximately 70 residues and represents the minimal region required for a methyl-CpG-binding protein to bind methylated DNA. As reports indicated, *RPL7* is a ribosome that catalyzes protein synthesis. It encodes a ribosomal protein and belongs to the L30P family of ribosomal proteins [28,29,30,31]. Regarding with the post-weaning gain trait, a GWAS study has reported three genes (*POL*, Complex Subunit 1 (*MSL1*) and Shisa Family Member 9 (*SHISA9*)) in sheep breeds [18,26]. Generally, all the genes discussed on the above have been playing a great role directly or indirectly in cell growth, embryonic development, differentiation of muscle tissue and nervous system. Genes, sheep breeds, phenotypic traits and location on the sheep genome are summarized in the table below (Table 1).

### 2.2. Genes Associated with Carcass and Fat Traits

Meat and carcass (including meat quality and carcass weight) are important phenotypic traits which are quite useful for human. In meat industry, meat quality is one of the most important meat traits that need more attention. Fatty acid composition is one of the factors that determine the effectiveness of meat quality because it affects human disease like obesity, diabetes, neurological diseases and cancers [32]. Besides, fatty acid composition determines the firmness and color of the meat [33], and shelf life of the meat also [34].

Genetic variants have been identified in calpastatin (*CAST*) gene, affecting meat quality and fatty acid composition in Chall and Zel sheep breeds [35]. *CAST* is an endogenous inhibitor protein acting on calpain Ca^2+^dependent cysteine proteinase. Moreover, PCR-SSCP study has identified genetic variants in leptin (*LEP*) gene associated with carcass and fat traits. These variants increase the weight of the carcass and body fat in Shal sheep, however, they decrease cold carcass and lean meat weight in Zel sheep [36]. Leptin has a regulatory function in sheep body weight, feed intake, growth and metabolic activities [37]. This showed that such genes have a great role in determining the quality of meat and carcass trait as well. Therefore, this should be future research focus to keep and produce high quality meat which is essential to mutton consumers.

PCR-SSCP study has identified genetic variants in myostatin (*MSTN*) gene which are related with meat traits. *MSTN* is a gene associated with meat production, and is characterized by decrease total lean meat and increase proportion of loin meat [38]. Genes, regulatory factor X associated ankyrin containing protein (*RFXANK*) and receptor-interacting serine/threonine-protein kinase 2 (*RIPK2*) have been also reported affecting post-weaning gain in sheep [26]. The *RIPK2* is important gene for modulation of innate and adaptive immune responses, whereas *RFXANK* regulates the protein making process to control the activity of *MHC* genes (Major histocompatibility complex class II) [39]. Furthermore, PCR-RFLP study has identified diacylglycerol acyltransferase1 (*DGAT1*) gene which controls carcass traits [40]. *DGAT1* is located on the endoplasmic reticulum membrane and is the only protein specific to triacylglycerol synthesis. It encodes the enzyme that catalyzes the reaction between diacylglycerol and acyl-CoA. Therefore, *DGAT1* controls the rate of triglycerides in adipocytes [41]. The uncoupling protein 1 (*UCP1*) is a gene belongs to the family of mitochondrial membrane carrier proteins (*MCPs*) and is predominantly expressed in brown adipose tissue. It plays major role in regulation of energy expenditure and protection against oxidative stress. A GWAS study in sheep reported that *UCP1* is a gene associated with subcutaneous carcass fat depth [42,43]. Genes, sheep breeds, phenotypic traits, and location on the sheep genome are summarized in the table below (Table 2). 

### 2.3. Genes Associated with Sheep Fertility Traits

Fertility traits such as ovulation rate, litter size, total lambs born, age at first lambing, stillbirth, and age at first puberty all have a significance effect and impact on the economy of sheep production industry. Of these, the first two traits are the most important and have high economic values [44]. The majority of sheep breeds give birth only singleton lambs and only a few give birth to two or more lambs [45]. This phenomenon is controlled by genetic and environmental factors [46]. Naturally, fecundity or fertility traits are genetically regulated by different genes with different effect in sheep [47]. A GWAS study has identified genes related with litter size, ovulation rate & sterility in sheep breeds. These genes include bone morphogenetic protein receptor IB (*BMPRIB*), bone morphogenetic protein 15 (*BMP15*) and growth differentiation factor 9 (*GDF9*) [48,49,50] *BMPRIB* influences the level of ovulation and litter size, whereas *BMP15* together with *GDF9* plays a significant role in follicle formation. Moreover, *BMP15* affects granulosa cells, theca cells and oocyte, whereas *GDF9* regulates ovarian follicle development. The gene, GDF9, is a protein that belongs to the *TGF-β* family [51,52,53].

In addition to the fecundity traits, there are also other important economic traits such as reproductive performance traits. A microsatellite genotyping study in prolactin receptor (*PRLR*) gene has revealed genetic variants related with reproductive performance in Herdwick, Rough Fells, and Dalesbred sheep breeds. *PRLR* is one of the member of class 1 cytokine receptors gene family [54]. Furthermore, a GWAS study has reported molecular variants in Cyclin B2 (*CCNB2*) and Solute Carrier Family 8 Member A3 (*SLC8A3*) genes associated with oocyte development in Chinese mongolian fat-tailed, German mutton merino and African white dorper sheep breeds [18]. The gene *SLC8A3* increases the availability of L-alanine and L-histidine for gap junctional transfer in oocytes [55]. Another GWAS study has identified a gene known as transmembrane protein 154 (*TMEM154*) associated with susceptibility to ovine lentivirus. The function of this gene is reducing lentivirus susceptibility [56,57]. Genes, sheep breeds, phenotypic traits, and location on sheep genome are prepared in the table below (Table 3).

### 2.4. Genes Associated with Sheep Milk Traits

Milk is one of the most essential mammalian production traits for human. The commonly known mammalian milk traits include milk yield, milk protein, milk fat, casein and lactose percentages. The production potential of these traits is mainly controlled by environmental factors and genetically by different genes. There are several molecular studies on milk traits in sheep. For instance, a PCR-RFLP study has revealed POU class 1 homeobox 1 (*POU1F1*) gene affecting milk production in Turkish sheep breed [60]. *POU1F1* is a member of POU containing transcription factor family which carries a POU DNA-binding domain. The POU DNA-binding domain is built from POU homedomainand POU-specific domain. The POU homedomainis required for DNA binding, whereas POU-specific domain for DNA-binding specifity and dimerization. *POU1F1* gene plays important role in regulating the function of growth hormone and prolactin genes. It is applicable for marker assisted selection [61].

Furthermore, a GWAS and genome-wide scan study have identified genes related with milk traits in different other sheep breeds. These genes include palmdelphin (*PALMD*) and ring finger protein 145 (*RFP145*) in Italian Altamurana sheep, and alpha-lactalbumin (*LALBA*) in Spanish Churra sheep. *PALMD* encoded for a cytosolic protein implicated in membrane dynamics, whereas *RFP145* gene involves in cellular processes [62,63]. The GH gene, which is encoded for growth hormone, affects not only growth traits but also milk traits. For instance, *GH1* is a gene associated with lower milk yield in Serrada Estrela sheep breed [64]. Generally, genetic variations on *GH* gene affects tissue expression and the function of the gene itself [65]. Genes, sheep breeds, phenotypic traits, and location on the sheep genome are summarized in the table below (Table 4).

### 2.5. Genes Affecting Wool, Coat Color and Horn Phenotypic Traits

Sheep wool is one of the important income generating traits and has been playing a main role in the global agricultural. The important wool traits that determine wool quality in sheep are fiber diameter, staple length, fineness dispersion and crimp [67]. In recent years, several candidate genes and molecular variants have been reported related with sheep wool traits. Genes, Keratin-Associated Protein (*KAP*) and Follistatin (*FST*) are among the genes reported to be associated with sheep wool. The *FST* gene was characterized for wool fiber diameter and wool crimp traits in chinese merino sheep (Xinjiang type) (CMXT). Ovine *FST* is a relatively small gene consisting of six exons located on chromosome 16. It is a single chain glycoprotein structurally unrelated with inhibin and activin [68]. In the same sheep breed, PCR-SSCP study was also found genetic variants in keratin-associated protein genes (*KAP6.1*, *KAP8.1*, *KAP8.2*, *KRTAP9-2*, and *KAP16.4*) related with wool quality. The keratin gene family regulates the function of wool, and development of hair follicles. Both *KAP* and *FST* genes can be used for marker assisted selection and sheep breeding program [68,69].

Coat color is one of the most important traits helped for breed identification and characterization [70], and other applications in goat, rabbit and sheep breeding [71]. Coat color variation is determined by biochemical function, distribution and availability of phaeomelanin and eumelanin pigments. Phaeomelanin and eumelanin are two different types of melanin responsible for pigment formation. Phaeomelanin produces red and yellow colors, whereas eumelanin for black color and brown phenotype [72]. Coat color is influenced by a number of genes participated in different stages of pigmentation (i.e., melanogenesis) [73]. To date, different genes and genetic variants have been identified to affect coat color. Agouti signaling protein (*ASIP*), melanocortin 1 receptor (*MC1R*), melanocyte inducing transcription factor (*MITF*), tyrosinase related protein-1 (*TYRP1*), and v-kit Hardy-Zuckerman 4 feline sarcoma viral oncogene homolog (*KIT*) are among the genes identified associated with coat color variation [74]. Of these, *ASIP* and *MC1R* plays significant role in determining coat color variation in sheep. *ASIP* has an antagonist function with *MC1R* on pigmentation making process. It affects the pigmentation activity by blocking the *α-MSH* receptor interaction and causes a pigment-type switching from eumelanin to pheomelanin [75]. A GWAS study has reported molecular variants in *MC1R* gene in Manchega and Rasa Aragonesa sheep [76], Chinese sheep [77], Brazilian Creole sheep [72] and Xalda sheep [78]. With the same approach, genetic variants have been reported in *ASIP* gene also in Dubian, Privorianp and Finn sheep breeds [75,76,77,78,79].

In ruminant, the horn phenotype is sexually selected by nature [80]. Generally speaking, both wild and domestic sheep have two horns, however, there are also another horn types. These are polyceraty, polled, scur and sometimes knob type. Polyceraty sheep has more than two horns (three horns, four horns, five horns and sometimes six horns), whereas polled sheep are those which has not horn. Polyceraty phenotypic trait was found in African, European and Asian sheep breeds [81,82,83]. A GWAS study has reported molecular variants in Relaxin/insulin-like family peptide receptor 2 (*RXFP2*) gene affecting polled phenotype in Herdwick, Rough Fells, Dalesbred and other sheep breeds [8,54,84,85,86]. *RXFP2* encodes a member of the GPCR (G protein-coupled, 7-transmembrane receptor) gene family. In contrast, metaxin 2 (*MTX2*) and Homeobox (*HOXD*) cluster (*HOXD9* & *HOXD3*) genes were identified affecting the polyceraty phenotype in Jacob, Navajo-Churro, Damara and Sishui Fur sheep breeds using GWAS. Both genes are responsible for horn existence and determining horn number in sheep [87,88,89,90]. Genes for wool, coat color and horn traits, and their respective location on the sheep genome are presented in the table below (Table 5)

## 3. Conclusions

The development of affordable sequencing technologies and software for large data analysis has contributed a lot to understanding the genetic mechanisms and genetic background of different phenotypic traits in sheep. The identification and characterization of candidate genes and genetic variants associated with economically important phenotypic traits is essential in animal breeding. Genetic improvement programs based on the detailed genetic information of the identified genes associated with traits of interest helps to increase production potential and productivity of domestic animals. Thus, the profitability and benefit of sheep producers as well as sheep product processing industries can increases. A number of genes and molecular variants associated with different phenotypic traits have been reported in sheep. Some of the reported genes affect more than one trait. Therefore, further functional verification study is important using novel genetic tools that integrate better computational and statistical analysis. In this review paper, genomic regions and related genes associated with economically important traits, as well as gene annotations, are discussed and summarized. This will be useful for genomic studies, genetic improvement programs, as well as breeding activities in sheep.

## Figures and Tables

**Table 1:** Description and chromosomal location of candidate genes associated with body weight and growth traits.

Genes	Chr	Position (bp)	Traits	Sheep Breed	Author(s)
*MEF2B*	5	3859638–3859738	BW,CG	Ujumqin sheep	[20]
*TRHDE*	3	108235641–108685027	BW,CG	Ujumqin sheep	[20]
DRB1*2001	20	25594470–25608591	WMW	Rambouillet&Columbia	[21]
*GH*	16	31832933–32000445	GT	Makui sheep	[24,25]
*CAPN*	12	25191495–25241603	BFW	Barki &Rahmani sheep	[13]
*MSTN*	2	118144443–118149433	GT	MRMT	[22,23]
*APOBR*	24	26065424–26069206	BMI	GMM, CMF and AWD	[18]
*FTO*	14	21524991–21953995	BMI	GMM, CMF and AWD	[18]
*TP53*	11	26935155–26939002	BS	Frizarta sheep	[19]
*GRM1*	8	70235268–70670365	PWG	SS, GMS and DS	[26]
*MBD5*	2	159880355–159937836	PWG	SS, GMS and DS	[26]
*UBR2*	20	16358563–16459598	PWG	SS, GMS and DS	[26]
*RPL7*	9	49443212–49445803	PWG	SS, GMS and DS	[26]
*SMC2*	2	18870300–18918858	PWG	SS, GMS and DS	[26]
*LHX3*	3	3138427–3148553	GT	HS, STHS, LFTS & TS	[11]
*LHX4*	12	59481470–59527642	GT	HS, STHS, LFTS & TS	[11]
*POL*	22	21493437–21500144	PWG	SS, GMS and DS	[26]
*MSL1*	11	39997471–40006992	PW	GMM, CMF and AWD	[18]
*SHISA9*	24	11921141–11946628	GM	SS, GMS and DS	[26]

BW, body weight; CG, chest girth; WMW, weaning & mature weights; GT, growth traits; BFW, Birth & final weight; BMI, body mass index; BS, Body size; PWG, post-weaning gain; PW, pre-weaning; GMM, German mutton merino; AWD, African white dorper; chr, chromosome; SS, Sunit sheep; DS, Dorper sheep; GMS, German mutton sheep; na, not available; CMF, Chinese Mongolian fat-tailed; MRMT, Madras Red, Mecheri, Texel.

**Table 2:** Description & chromosomal location of genes related with carcass and body fat traits.

Genes	Chr	Position (bp)	Traits	Sheep Breed	Author(s)
*DGAT1*	9	13566142–13575279	BFTW	Lori-Bakhtiari & Zel	[40]
*UCP1*	17	16847682–16853677	SCFD	NZ, Romney & Suffolk	[43]
	17	16847682–16853677	TLLM	NZ, Romney & Suffolk	[43]
	17	16847682–16853677	TLHM	NZ, Romney & Suffolk	[43]
*LEP*	4	92508289–92522182	CW	Shal sheep	[36]
	4	92508289–92522182	FP	Shal sheep	[36]
	4	92508289–92522182	TBFW	Shal sheep	[36]
	4	92508289–92522182	FP	Zel sheep	[36]
	4	92508289–92522182	SW	Zel sheep	[36]
	4	92508289–92522182	CW	Zel sheep	[36]
	4	92508289–92522182	LMW	Zel sheep	[36]
	4	92508289–92522182	WW	Zandi sheep	[36]
*CAST*	5	93354399–93484087	MQ	Chall Iranian sheep	[35]
*CAST*	5	93354399–93484087	FAP	Zel Iranian sheep	[35]
*RFXANK*	5	3825886–3830327	GM	SS, GMS and DS	[26]
*RIPK2*	9	86491784–86524759	GM	SS, GMS and DS	[26]
*MSTN*	2	118144443–118149433	MY	New Zealand, Romney	[38]

BFTW, back fat thickness and weight; SCFD, Subcutaneous Carcass Fat depth; TLLM, total lean loin meat; TLHM, total lean hind-leg meat; CW, carcass weight; FTP, fat-tail percent; TBFW, total body fat weight; SW, slaughter weight; LMW, lean meat weight; WW, weaning weight; MQ, meat quality; FAP, fatty acid profiles; GM, growth and meat; MPQ, meat production and quality; GMM, German mutton merino; AWD, African white dorper; NZ, New zealand; chr, chromosome; SS, Sunit sheep; DS, Dorper sheep; GMS, German mutton sheep; na, not available; CMF, Chinese Mongolian fat-tailed; MY, meat yield.

**Table 3:** Candidate genes for fertility traits and their location.

Genes	Chr	Position (bp)	Traits	Sheep Breed	Author(s)
*PRLR*	16	38969273–39028126	RP	Herdwick & RFD	[54]
*TMEM154*	17	4832841–4882002	ILV	Herdwick & RFD	[54,56]
*CCNB2*	7	48194193–48217973	OD	GMM, CMF & AWD	[18]
*SLC8A3*	7	78697982–78837399	OD	GMM, CMF & AWD	[18]
*GDF9*	5	41841034–41843517	ORS	Cambridge & Belclare	[50]
*BMP15*	X	50970938–50977454	ORS	Cambridge & Belclare	[48,50]
*BMPRIB*	6	29361947–29448079	ORS	Lacaune Sheep	[49,58]
*BMPRIB*	7	29361947–29448079	LS	Han sheep	[59]
*BMP15*	X	50970938–50977454	LS	Han sheep	[59]

RP, reproductive performance; ILV, Infection to the lentivirus; OD, Oocyte development; ORS, Ovulation rate & sterility; LS, Litter size; GMM, German mutton merino; AWD, African white dorper; chr, chromosome; na, not available; FD, Fells & Dalesbred; CMF, Chinese Mongolian fat-tailed.

**Table 4:** Genes for milk yield traits.

Gene	Chr	Position (bp)	Traits	Sheep Breed	Author(s)
*POU1F1*	1	154027868–154043592	MY	Turkish sheep	[60]
*RFP145*	na	na	MY	Italian Altamurana	[62]
*LALBA*	3	137390403–137392415	MP	Spanish Churra	[63]
*GH1*	11	47540169–47541799	milk	Serrada Estrela	[66]
*PALMD*	na	na	MY	Italian Altamurana	[62]

MY, milk yield; MP, milk production; GMM, German mutton merino; AWD, African white dorper; chr, chromosome; na, not available; CMF, Chinese Mongolian fat-tailed.

**Table 5:** Description and chromosomal location of genes associated with wool and coat color traits.

Gene	Chr	Position (bp)	Traits	Sheep Breed	Author
*KAP6.1*	na	na	wool	Merino (Xinjiang type)	[69]
*KAP8.1*	1	123013704–123013892	wool	Merino (Xinjiang type)	[69]
*KAP8.2*	na	na	wool	Merino (Xinjiang type)	[69]
*KRTAP9-2*	na	na	wool	Merino (Xinjiang type)	[69]
*KAP16.4*	na	na	wool	Merino (Xinjiang type)	[69]
*FST*	16	25630860–25636124	WQ	CMS (Junken Type)	[68]
*MC1R*	14	14231363–14232541	color	MRA	[76]
*ASIP*		na	color	Dubian &Privorianp	[75]
*RXFP2*	10	29454677–29502617	horn	Herdwick & RFD	[54]
*HOXD*	2	132.0–133.1 Mb	HE	NDS	[87,88,90]
*MTX2*	2	131.9–32.6 Mb	HN	NDS	[87,88,90]

WG, wool & growth; WQ, wool quality; HT, hair thickness; chr, chromosome; na, not available; CMS, Chinese Merino Sheep; RFD, Rough Fells & Dalesbred; MRA, Manchega & Rasa Aragonesa; CMF, Chinese Mongolian fat-tailed, HE, horn existence; HN, horn number; NDS, Navajo-churro, damara and Sishui Fur.

## References

[1] Montossi F., Font-i-Furnols M., Del Campo M., San Julián R., Brito G., Sañudo C. (2013). Sustainable sheep production and consumer preference trends: Compatibilities, contradictions, and unresolved dilemmas. Meat Sci..

[2] Miles C., Wayne M. (2008). Quantitative Trait Locus (QTL) Analysis.

[3] Zhang H., Wang Z., Wang S., Li H. (2012). Progress of genome wide association study in domestic animals. J. Anim. Sci. Biotechnol..

[4] Hirschhorn J.N., Daly M.J. (2005). Genome-wide association studies for common diseases and complex traits. Nat. Rev. Genet..

[5] Xu S.-S., Li M.-H. (2017). Recent advances in understanding genetic variants associated with economically important traits in sheep (*Ovis aries*) revealed by high-throughput screening technologies. Front. Agric. Sci. Eng..

[6] Ozaki K., Ohnishi Y., Iida A., Sekine A., Yamada R., Tsunoda T., Sato H., Sato H., Hori M., Nakamura Y. (2002). Functional SNPs in the lymphotoxin-α gene that are associated with susceptibility to myocardial infarction. Nat. Genet..

[7] Klein R.J., Zeiss C., Chew E.Y., Tsai J.-Y., Sackler R.S., Haynes C., Henning A.K., SanGiovanni J.P., Mane S.M., Mayne S.T. (2005). Complement factor H polymorphism in age-related macular degeneration. Science.

[8] Johnston S.E., Mcewan J.C., Pickering N.K., Kijas J.W., Beraldi D., Pilkington J.G., Pemberton J.M., Slate J. (2011). Genome-wide association mapping identifies the genetic basis of discrete and quantitative variation in sexual weaponry in a wild sheep population. Mol. Ecol..

[9] Daw E.W., Heath S.C., Lu Y. (2005). Single-Nucleotide Polymorphism Versus Microsatellite Markers in a Combined Linkage and Segregation Analysis of a Quantitative Trait.

[10] Hong L., Dumond M., Tsugawa S., Sapala A., Routier-Kierzkowska A.-L., Zhou Y., Chen C., Kiss A., Zhu M., Hamant O. (2016). Variable cell growth yields reproducible organ development through spatiotemporal averaging. Dev. Cell.

[11] Zhao H., He S., Zhu Y., Cao X., Luo R., Cai Y., Xu H., Sun X. (2017). A novel 29 bp insertion/deletion (indel) variant of the LHX3 gene and its influence on growth traits in four sheep breeds of various fecundity. Arch. Anim. Breed..

[12] Park S., Mullen R.D., Rhodes S.J. (2013). Cell-specific actions of a human LHX3 gene enhancer during pituitary and spinal cord development. Mol. Endocrinol..

[13] Mahrous K., Hassanane M., Shafey H., Mordy M.A., Rushdi H. (2016). Association between single nucleotide polymorphism in ovine Calpain gene and growth performance in three Egyptian sheep breeds. J. Genet. Eng. Biotechnol..

[14] Arora R., Yadav H.S., Yadav D.K. (2014). Identification of novel single nucleotide polymorphisms in candidate genes for mutton quality in Indian sheep. Anim. Mol. Breed..

[15] Kumar N., Jayashankar M., Ramakrishnappa N., Nagaraja C., Fairoze N., Satyanarayana K. (2015). Genetic polymorphism of ovine calpain gene in Bandur sheep. Int. J. Sci. Environ. Technol..

[16] Huff-Lonergan E., Mitsuhashi T., Beekman D.D., Parrish F.C., Olson D.G., Robson R.M. (1996). Proteolysis of specific muscle structural proteins by µ-calpain at low pH and temperature is similar to degradation in postmortem bovine muscle. J. Anim. Sci..

[17] Goll D.E., Thompson V.F., Taylor R.G., Zalewska T. (1992). Is calpain activity regulated by membranes and autolysis or by calcium and calpastatin?. Bioessays.

[18] Wang H., Zhang L., Cao J., Wu M., Ma X., Liu Z., Liu R., Zhao F., Wei C., Du L. (2015). Genome-wide specific selection in three domestic sheep breeds. PLoS ONE.

[19] Kominakis A., Hager-Theodorides A.L., Zoidis E., Saridaki A., Antonakos G., Tsiamis G. (2017). Combined GWAS and ‘guilt by association’-based prioritization analysis identifies functional candidate genes for body size in sheep. Genet. Sel. Evol..

[20] Zhang L., Ma X., Xuan J., Wang H., Yuan Z., Wu M., Liu R., Zhu C., Wei C., Zhao F. (2016). Identification of MEF2B and TRHDE gene polymorphisms related to growth traits in a new Ujumqin sheep population. PLoS ONE.

[21] Cinar M.U., Mousel M.R., Herrmann-Hoesing L.M., Taylor J.B., White S.N. (2016). Ovar-DRB1 haplotypes * 2001 and * 0301 are associated with sheep growth and ewe lifetime prolificacy. Gene.

[22] Broad T., Glass B., Greer G., Robertson T., Bain W., Lord E., McEwan J., Peterson S. (2000). Search for a locus near to myostatin that increases muscling in Texel sheep in New Zealand. Proceedings of the New Zealand Society of Animal Production, Hamilton, New Zealand, 26–29 June 2000.

[23] Sahu A.R., Jeichitra V., Rajendran R., Raja A. (2017). Polymorphism in exon 3 of myostatin (MSTN) gene and its association with growth traits in Indian sheep breeds. Small Rumin. Res..

[24] Moradian C., Mohamadi N., Sheshdeh S., Hajihosseinlo A., Ashrafi F. (2013). Effects of genetic polymorphismat the growth hormone gene on growth traits in Makooei sheep. Eur. J. Exp. Biol..

[25] McMahon C., Radcliff R., Lookingland K., Tucker H. (2001). Neuroregulation of growth hormone secretion in domestic animals. Domest. Anim. Endocrinol..

[26] Zhang L., Liu J., Zhao F., Ren H., Xu L., Lu J., Zhang S., Zhang X., Wei C., Lu G. (2013). Genome-wide association studies for growth and meat production traits in sheep. PLoS ONE.

[27] Strunnikov A.V., Hogan E., Koshland D. (1995). SMC2, a Saccharomyces cerevisiae gene essential for chromosome segregation and condensation, defines a subgroup within the SMC family. Genes Dev..

[28] Cavanagh C.R., Jonas E., Hobbs M., Thomson P.C., Tammen I., Raadsma H.W. (2010). Mapping Quantitative Trait Loci (QTL) in sheep. III. QTL for carcass composition traits derived from CT scans and aligned with a meta-assembly for sheep and cattle carcass QTL. Genet. Sel. Evol..

[29] Talkowski M.E., Rosenfeld J.A., Blumenthal I., Pillalamarri V., Chiang C., Heilbut A., Ernst C., Hanscom C., Rossin E., Lindgren A.M. (2012). Sequencing chromosomal abnormalities reveals neurodevelopmental loci that confer risk across diagnostic boundaries. Cell.

[30] Du Y., Liu B., Guo F., Xu G., Ding Y., Liu Y., Sun X., Xu G. (2012). The essential role of Mbd5 in the regulation of somatic growth and glucose homeostasis in mice. PLoS ONE.

[31] Cukier H.N., Lee J.M., Ma D., Young J.I., Mayo V., Butler B.L., Ramsook S.S., Rantus J.A., Abrams A.J., Whitehead P.L. (2012). The expanding role of MBD genes in autism: Identification of a MECP2 duplication and novel alterations in MBD5, MBD6, and SETDB1. Autism Res..

[32] Ntambi J.M., Miyazaki M. (2004). Regulation of stearoyl-CoA desaturases and role in metabolism. Prog. Lipid Res..

[33] Webb E., O’neill H. (2008). The animal fat paradox and meat quality. Meat Sci..

[34] Renerre M. (2000). Oxidative processes and myoglobin. Antioxid. Muscle Foods.

[35] Aali M., Moradi-Shahrbabak H., Moradi-Shahrbabak M., Sadeghi M., Yousefi A.R. (2017). Association of the calpastatin genotypes, haplotypes, and SNPs with meat quality and fatty acid composition in two Iranian fat-and thin-tailed sheep breeds. Small Rumin. Res..

[36] Barzehkar R., Salehi A., Mahjoubi F. (2009). Polymorphisms of the ovine leptin gene and its association with growth and carcass traits in three Iranian sheep breeds. Iran. J. Biotechnol..

[37] Frühbeck G., Jebb S., Prentice A. (1998). Leptin: Physiology and pathophysiology. Clin. Physiol..

[38] Hickford J., Forrest R., Zhou H., Fang Q., Han J., Frampton C., Horrell A. (2010). Polymorphisms in the ovine myostatin gene (MSTN) and their association with growth and carcass traits in New Zealand Romney sheep. Anim. Genet..

[39] Krawczyk M., Masternak K., Zufferey M., Barras E., Reith W. (2005). New functions of the major histocompatibility complex class II-specific transcription factor RFXANK revealed by a high-resolution mutagenesis study. Mol. Cell. Biol..

[40] Mohammadi H., Shahrebabak M.M., Sadeghi M. (2013). Association between single nucleotide polymorphism in the ovine DGAT1 gene and carcass traits in two Iranian sheep breeds. Anim. Biotechnol..

[41] Mayorek N., Grinstein I., Bar-Tana J. (1989). Triacylglycerol synthesis in cultured rat hepatocytes: The rate-limiting role of diacylglycerol acyltransferase. Eur. J. Biochem..

[42] Cannon B., Nedergaard J. (2004). Brown adipose tissue: Function and physiological significance. Physiol. Rev..

[43] Yang G., Forrest R., Zhou H., Hodge S., Hickford J. (2014). Genetic variation in the ovine uncoupling protein 1 gene: Association with carcass traits in N ew Z ealand (NZ) R omney sheep, but no association with growth traits in either NZ R omney or NZ S uffolk sheep. J. Anim. Breed. Genet..

[44] Chen H.Y., Shen H., Jia B., Zhang Y.S., Wang X.H., Zeng X.C. (2015). Differential gene expression in ovaries of Qira black sheep and Hetian sheep using RNA-Seq technique. PLoS ONE.

[45] Miao X., Luo Q., Zhao H., Qin X. (2016). Co-expression analysis and identification of fecundity-related long non-coding RNAs in sheep ovaries. Sci. Rep..

[46] Miao X., Luo Q. (2013). Genome-wide transcriptome analysis between small-tail Han sheep and the Surabaya fur sheep using high-throughput RNA sequencing. Reproduction.

[47] Drouilhet L., Lecerf F., Bodin L., Fabre S., Mulsant P. (2009). Fine mapping of the FecL locus influencing prolificacy in Lacaune sheep. Anim. Genet..

[48] Galloway S.M., McNatty K.P., Cambridge L.M., Laitinen M.P., Juengel J.L., Jokiranta T.S., McLaren R.J., Luiro K., Dodds K.G., Montgomery G.W. (2000). Mutations in an oocyte-derived growth factor gene (BMP15) cause increased ovulation rate and infertility in a dosage-sensitive manner. Nat. Genet..

[49] Souza C., MacDougall C., Campbell B., McNeilly A., Baird D. (2001). The Booroola (FecB) phenotype is associated with a mutation in the bone morphogenetic receptor type 1 B (BMPR1B) gene. J. Endocrinol..

[50] Hanrahan J.P., Gregan S.M., Mulsant P., Mullen M., Davis G.H., Powell R., Galloway S.M. (2004). Mutations in the genes for oocyte-derived growth factors GDF9 and BMP15 are associated with both increased ovulation rate and sterility in Cambridge and Belclare sheep (*Ovis aries*). Biol. Reprod..

[51] Davis G., Montgomery G., Allison A., Kelly R., Bray A. (1982). Segregation of a major gene influencing fecundity in progeny of Booroola sheep. N. Z. J. Agric. Res..

[52] Yan C., Wang P., DeMayo J., DeMayo F.J., Elvin J.A., Carino C., Prasad S.V., Skinner S.S., Dunbar B.S., Dube J.L. (2001). Synergistic roles of bone morphogenetic protein 15 and growth differentiation factor 9 in ovarian function. Mol. Endocrinol..

[53] McNatty K.P., Galloway S.M., Wilson T., Smith P., Hudson N.L., O’Connell A., Bibby A.H., Heath D.A., Davis G.H., Hanrahan J.P. (2005). Physiological Effects of Major Genes Affecting Ovulation Rate in Sheep.

[54] Bowles D., Carson A., Isaac P. (2014). Genetic distinctiveness of the Herdwick sheep breed and two other locally adapted hill breeds of the UK. PLoS ONE.

[55] Eppig J.J., Pendola F.L., Wigglesworth K., Pendola J.K. (2005). Mouse oocytes regulate metabolic cooperativity between granulosa cells and oocytes: Amino acid transport. Biol. Reprod..

[56] Heaton M.P., Clawson M.L., Chitko-Mckown C.G., Leymaster K.A., Smith T.P., Harhay G.P., White S.N., Herrmann-Hoesing L.M., Mousel M.R., Lewis G.S. (2012). Reduced lentivirus susceptibility in sheep with TMEM154 mutations. PLoS Genet..

[57] White S.N., Mousel M.R., Herrmann-Hoesing L.M., Reynolds J.O., Leymaster K.A., Neibergs H.L., Lewis G.S., Knowles D.P. (2012). Genome-wide association identifies multiple genomic regions associated with susceptibility to and control of ovine lentivirus. PLoS ONE.

[58] Bodin L., Di Pasquale E., Fabre S., Bontoux M., Monget P., Persani L., Mulsant P. (2007). A novel mutation in the bone morphogenetic protein 15 gene causing defective protein secretion is associated with both increased ovulation rate and sterility in Lacaune sheep. Endocrinology.

[59] Chu M., Liu Z., Jiao C., He Y., Fang L., Ye S., Chen G., Wang J. (2007). Mutations in BMPR-IB and BMP-15 genes are associated with litter size in Small Tailed Han sheep (*Ovis aries*). J. Anim. Sci..

[60] Ozmen O., Kul S., Unal E.O. (2014). Polymorphism of sheep POU1F1 gene exon 6 and 3′UTR region and their association with milk production traits. Iran. J. Vet. Res..

[61] Lan X., Li M., Chen H., Zhang L., Jing Y., Wei T., Ren G., Wang X., Fang X., Zhang C. (2009). Analysis of caprine pituitary specific transcription factor-1 gene polymorphism in indigenous Chinese goats. Mol. Biol. Rep..

[62] Moioli B., Scatà M.C., Steri R., Napolitano F., Catillo G. (2013). Signatures of selection identify loci associated with milk yield in sheep. BMC Genet..

[63] García-Gámez E., Gutiérrez-Gil B., Sahana G., Sánchez J.-P., Bayón Y., Arranz J.-J. (2012). GWA analysis for milk production traits in dairy sheep and genetic support for a QTN influencing milk protein percentage in the LALBA gene. PLoS ONE.

[64] do Rosário Marques M., Santos I.C., Carolino N., Belo C.C., Renaville R., Cravador A. (2006). Effects of genetic polymorphisms at the growth hormone gene on milk yield in Serra da Estrela sheep. J. Dairy Res..

[65] Dettori M.L., Pazzola M., Pira E., Paschino P., Vacca G.M. (2015). The sheep growth hormone gene polymorphism and its effects on milk traits. J. Dairy Res..

[66] Vacca G., Dettori M., Balia F., Luridiana S., Mura M., Carcangiu V., Pazzola M. (2013). Sequence polymorphisms at the growth hormone GH1/GH2-N and GH2-Z gene copies and their relationship with dairy traits in domestic sheep (*Ovis aries*). Mol. Biol. Rep..

[67] Wang Z., Zhang H., Yang H., Wang S., Rong E., Pei W., Li H., Wang N. (2014). Genome-wide association study for wool production traits in a Chinese Merino sheep population. PLoS ONE.

[68] Ma G.-W., Chu Y.-K., Zhang W.-J., Qin F.-Y., Xu S.-S., Yang H., Rong E.-G., Du Z.-Q., Wang S.-Z., Li H. (2017). Polymorphisms of FST gene and their association with wool quality traits in Chinese Merino sheep. PLoS ONE.

[69] Sulayman A., Mamat A., Taursun M., Huang X.-X., Tian K., Tian Y., Xu X., Fu X. (2017). Identification of Polymorphisms and Association of Five KAP Genes with Sheep Wool Traits. Asian Australas. J. Anim. Sci..

[70] Norris B.J., Whan V.A. (2008). A gene duplication affecting expression of the ovine ASIP gene is responsible for white and black sheep. Genome Res..

[71] Fontanesi L., Beretti F., Riggio V., Dall’Olio S., Calascibetta D., Russo V., Portolano B. (2010). Sequence characterization of the melanocortin 1 receptor (MC1R) gene in sheep with different coat colours and identification of the putative e allele at the ovine Extension locus. Small Rumin. Res..

[72] Hepp D., Gonçalves G., Moreira G., Freitas T., Martins C., Weimer T., Passos D. (2012). Identification of the e allele at the Extension locus (MC1R) in Brazilian Creole sheep and its role in wool color variation. Genet. Mol. Res..

[73] Hoekstra H.E. (2006). Genetics, development and evolution of adaptive pigmentation in vertebrates. Heredity.

[74] Cieslak M., Reissmann M., Hofreiter M., Ludwig A. (2011). Colours of domestication. Biol. Rev..

[75] Fontanesi L., Rustempašić A., Brka M., Russo V. (2012). Analysis of polymorphisms in the agouti signalling protein (ASIP) and melanocortin 1 receptor (MC1R) genes and association with coat colours in two Pramenka sheep types. Small Rumin. Res..

[76] Kijas J., Serrano M., McCulloch R., Li Y., Salces Ortiz J., Calvo J., Pérez-Guzmán M., Consortium I.S.G. (2013). Genomewide association for a dominant pigmentation gene in sheep. J. Anim. Breed. Genet..

[77] Yang G.-L., Fu D.-L., Lang X., Wang Y.-T., Cheng S.-R., Fang S.-L., Luo Y.-Z. (2013). Mutations in MC1R gene determine black coat color phenotype in Chinese sheep. Sci. World J..

[78] Royo L.J., Alvarez I., Arranz J., Fernández I., Rodríguez A., Pérez-Pardal L., Goyache F. (2008). Differences in the expression of the ASIP gene are involved in the recessive black coat colour pattern in sheep: Evidence from the rare Xalda sheep breed. Anim. Genet..

[79] Li M., Tiirikka T., Kantanen J. (2014). A genome-wide scan study identifies a single nucleotide substitution in ASIP associated with white versus non-white coat-colour variation in sheep (*Ovis aries*). Heredity.

[80] Johnston S.E., Gratten J., Berenos C., Pilkington J.G., Clutton-Brock T.H., Pemberton J.M., Slate J. (2013). Life history trade-offs at a single locus maintain sexually selected genetic variation. Nature.

[81] Maiwashe A., Blackburn H. (2004). Genetic diversity in and conservation strategy considerations for Navajo Churro sheep. J. Anim. Sci..

[82] Dýrmundsson Ó.R. (2005). Four-hornedness; a rare peculiarity still found in Icelandic sheep. Icel. Sheep Breed. N. Am. Newsl..

[83] Sponenberg D., Taylor C. (2009). Navajo-Churro sheep and wool in the United States. Anim. Genet. Resour. Resour. Génétiques Anim. Recur. Genéticos Anim..

[84] Wiedemar N., Drögemüller C. (2015). A 1.8-kb insertion in the 3′-UTR of RXFP2 is associated with polledness in sheep. Anim. Genet..

[85] Dominik S., Henshall J., Hayes B. (2012). A single nucleotide polymorphism on chromosome 10 is highly predictive for the polled phenotype in Australian Merino sheep. Anim. Genet..

[86] Kijas J.W., Lenstra J.A., Hayes B., Boitard S., Neto L.R.P., San Cristobal M., Servin B., McCulloch R., Whan V., Gietzen K. (2012). Genome-wide analysis of the world’s sheep breeds reveals high levels of historic mixture and strong recent selection. PLoS Biol..

[87] He X., Zhou Z., Pu Y., Chen X., Ma Y., Jiang L. (2016). Mapping the four-horned locus and testing the polled locus in three Chinese sheep breeds. Anim. Genet..

[88] Greyvenstein O.C., Reich C., van Marle-Koster E., Riley D., Hayes B. (2016). Polyceraty (multi-horns) in Damara sheep maps to ovine chromosome 2. Anim. Genet..

[89] Kijas J.W., Hadfield T., Naval Sanchez M., Cockett N. (2016). Genome-wide association reveals the locus responsible for four-horned ruminant. Anim. Genet..

[90] Ren X., Yang G.-L., Peng W.-F., Zhao Y.-X., Zhang M., Chen Z.-H., Wu F.-A., Kantanen J., Shen M., Li M.-H. (2016). A genome-wide association study identifies a genomic region for the polycerate phenotype in sheep (*Ovis aries*). Sci. Rep..

